# Over-Expression of TRESK K^+^ Channels Reduces the Excitability of Trigeminal Ganglion Nociceptors

**DOI:** 10.1371/journal.pone.0087029

**Published:** 2014-01-23

**Authors:** Zhaohua Guo, Yu-Qing Cao

**Affiliations:** Washington University Pain Center and Department of Anesthesiology, Washington University School of Medicine, St. Louis, Missouri, United States of America; University of Kentucky Medical Center, United States of America

## Abstract

TWIK-related spinal cord K^+^ (TRESK) channel is abundantly expressed in trigeminal ganglion (TG) and dorsal root ganglion neurons and is one of the major background K^+^ channels in primary afferent neurons. Mutations in TRESK channels are associated with familial and sporadic migraine. In rats, both chronic nerve injury and inflammation alter the expression level of TRESK mRNA. Functional studies indicate that reduction of endogenous TRESK channel activity results in hyper-excitation of primary afferent neurons, suggesting that TRESK is a potential target for the development of new analgesics. However, whether and how enhancing TRESK channel activity would decrease the excitability of primary afferent neurons has not been directly tested. Here, we over-expressed TRESK subunits in cultured mouse TG neurons by lipofectamine-mediated transfection and investigated how this altered the membrane properties and the excitability of the small-diameter TG population. To account for the heterogeneity of neurons, we further divided small TG neurons into two groups, based on their ability to bind to fluorescently-labeled isolectin B (IB4). The transfected TG neurons showed a 2-fold increase in the level of TRESK proteins. This was accompanied by a significant increase in the fraction of lamotrigine-sensitive persistent K^+^ currents as well as the size of total background K^+^ currents. Consequently, both IB4-positive and IB4-negative TG neurons over-expressing TRESK subunits exhibited a lower input resistance and a 2-fold increase in the current threshold for action potential initiation. IB4-negative TG neurons over-expressing TRESK subunits also showed a significant reduction of the spike frequency in response to supra-threshold stimuli. Importantly, an increase in TRESK channel activity effectively inhibited capsaicin-evoked spikes in TG neurons. Taken together, our results suggest that potent and specific TRESK channel openers likely would reduce the excitability of primary afferent neurons and therefore are potential therapeutics for the treatment of migraine and other chronic pain symptoms.

## Introduction

Two-pore domain K^+^ (K_2P_) channels mediate the background (also called leak) K^+^ currents that determine the resting membrane potential (V_rest_) of all cells. TWIK-related spinal cord K^+^ (TRESK) channel belongs to the K_2P_ channel family and is abundantly expressed in primary afferent neurons in the trigeminal ganglion (TG) and dorsal root ganglion (DRG) [1]–[5]. The activity of TRESK channels can be modulated by pH, volatile anesthetics, intracellular Ca^2+^ as well as other signaling pathways [2], [4], [6]–[8]. Previous studies indicate that TRESK is one of the major background K^+^ channels in primary afferent neurons and controls neuronal excitability in both normal and disease settings [9], [10]. In rats, both chronic nerve injury and inflammation alter the expression level of TRESK mRNA [11], [12]. DRG neurons from the TRESK functional knockout mice exhibit a reduction of background K^+^ current as well as a decrease in rheobase, the minimum current threshold required to elicit an action potential (AP), relative to wild-type neurons [9]. The inhibition of TRESK and other K_2P_ channels by sanshool, the active ingredient of Sichuan pepper, robustly increases the firing of subpopulations of rapidly-adapting mechanoreceptors and C fibers, and is proposed as the physiological basis of tingling paresthesia [1], [13]. Hindpaw injection of sanshool derivative IBA elicits nocifensive behavior [12]. Knocking down of TRESK channel expression by siRNA increases the sensitivity to painful pressure [12].

Recently, a frameshift mutation in human KCNK18 gene encoding the TRESK channel subunits has been linked to migraine with aura in a large pedigree [3]. The mutation results in the truncation of TRESK protein in the second transmembrane domain. We have shown that the truncated TRESK subunits exert a dominant-negative effect on currents through the endogenous TRESK channels in TG neurons. Consequently, neurons expressing mutant TRESK subunits exhibit a higher input resistance (R_in_), a lower rheobase as well as a higher spike frequency in response to supra-threshold stimuli, indicating that the mutation resulted in hyper-excitability of TG neurons [14].

These functional studies, along with the abundant expression of TRESK channels in primary afferent neurons, suggest that TRESK might be a potential therapeutic target for the treatment of both acute and chronic pain syndromes. Indeed, over-expression of TRESK subunits in DRG neurons inhibits capsaicin-evoked substance P release and attenuates nerve injury-induced mechanical allodynia [15], [16]. Here, we conducted proof-of-concept experiments to test whether increasing TRESK channel activity in TG neurons might be used as a potential treatment for migraine headache and other orofacial pain. To this end, we over-expressed TRESK subunits in cultured mouse TG neurons. This resulted in an increase in background K^+^ currents, a decrease of R_in_, and ultimately a significant decrease in the excitability of TG neurons. Importantly, over-expression of TRESK subunits inhibited capsaicin-evoked spikes in TG neurons, suggesting that a TRESK-specific channel opener may exhibit an analgesic effect via reducing the excitability of primary afferent neurons.

## Materials and Methods

### Ethics Statement

All procedures in this study were approved by the Animal Studies Committee at Washington University in St. Louis. The breeders were maintained on a 12-h light/dark cycle with constant temperature (23–24°C), humidity (45–50%) as well as food and water *ad libitum* at the animal facility of Washington University in St. Louis.

### Wild-type Mouse TRESK Constructs

The coding region of the mouse TRESK K^+^ channel (encoded by the kcnk18 gene) was PCR amplified from a plasmid purchased from Open Biosystems and was cloned into the plasmid pIRES2-DsRed-Express2 (Clontech) to generate the construct wtTRESK-IRES-DsRed [14]. The mCherry-tagged wild-type TRESK construct (mCherry-TRESK) was generated by fusing wild-type TRESK cDNA in frame at the C-terminus of mCherry coding region. All PCR-generated cDNA fragments and linker regions were completely sequenced to make sure that no mutations were introduced. In all constructs, the TRESK coding regions were inserted downstream of the CMV promoter.

### Cell Culture and Transfection

Human embryonic kidney 293T (HEK293T) cells (ATCC) were maintained in 6-well plates in DMEM with 10% fetal bovine serum (FBS) and were transfected with Lipofectamine 2000 (Invitrogen). One day post-transfection, cells were seeded on matrigel-coated coverslips. Cells were used for patch clamp recording 2–3 days post-transfection.

TG neurons were cultured from newborn CD-1 mice of either sex. TG tissues were collected from postnatal day 1 pups and were treated with 5 mg/ml trypsin for 15 min. Neurons were dissociated by triturating with fire-polished glass pipettes and were seeded on matrigel-coated coverslips. The MEM-based culture medium contained 5% FBS, 25 ng/ml nerve growth factor and 10 ng/ml glial cell line-derived neurotrophic factor and was replaced every 3 days. Cultured TG neurons from neonatal mice usually grow 1–3 processes from soma by 2 days *in vitro* (DIV). Longer culture time (3–6 DIV) does not increase the number of processes or the thickness of the existing processes from soma, but significantly increase the length and the number of branches of individual processes.

TG neurons were transfected with plasmids encoding mCherry and mCherry-TRESK proteins at 1 DIV using lipofectamine 2000 (Invitrogen), respectively. Transfected neurons were identified by mCherry fluorescence and were recorded between 3–6 DIV. The processes of the transfected neurons would contribute to the space clamp error. It is unlikely that the prolonged culture time would exacerbate the space clamp error, as we did not find significant differences between early (3 DIV) and late (6 DIV) recordings within individual experimental groups.

### Immunostaining and Image Analysis

Two days post-transfection, TG neurons were washed with phosphate-buffered saline (PBS) and were fixed by 4% formaldehyde for 5 min followed by PBS wash. The coverslips were incubated in blocking buffer (PBS with 10% normal goat serum and 0.1% triton X-100) for 1 hr and were then incubated with a mouse polyclonal TRESK antibody (1∶1000, [14]) in blocking buffer at 4°C overnight. Following 3 washes by the blocking buffer (20 min each), the coverslips were incubated with the AlexaFluor 488-conjugated goat anti-mouse secondary antibody (Invitrogen, 1∶2000) in blocking buffer for 1 hr and were washed again 3 times in PBS. The coverslips were mounted with Crystal/Mount medium and stored at 4°C.

Transfected cells were identified by the mCherry fluorescence. The differential interference contrast (DIC) and fluorescent images were captured through a 40x objective (N.A. 1.3) on a Nikon TE2000S inverted epifluorescence microscope equipped with a CoolSnap HQ^2^ camera (Photometrics). SimplePCI software (Hamamatsu) was used for image analysis. To measure the level of TRESK-immunoreactivity (TRESK-ir), the DIC image of each cell was specified as a region of interest (ROI). The intensity of AlexaFluor 488 signal was determined on a pixel-by-pixel basis and was averaged for each ROI. For each image captured, the mean intensity of the cell-free regions was taken as the background level and subtracted from the mean intensity in each ROI.

To test the specificity of the TRESK antibody, we incubated the antisera (1∶1000 dilution) with 1 µg of antigen (∼1∶10 antibody to antigen molar ratio) in blocking buffer at 4°C overnight. After centrifugation at 15,000 g for 30 min to remove the antigen-antibody complex, the supernatant was applied to cultured TG neurons for immunostaining. The signal was compared to that of cultured TG neurons stained with the secondary antibody only.

### Electrophysiology

Transfected cells were identified by the DsRed and/or mCherry fluorescence. Whole-cell patch-clamp recordings were performed at room temperature with a MultiClamp 700B amplifier (Molecular Devices). The recording chamber was perfused with Tyrode solution (0.5 ml/min) containing (in mM): 130 NaCl, 2 KCl, 2 CaCl_2_, 2 MgCl_2_, 25 HEPES, 30 glucose, pH 7.3 with NaOH, 310 mOsm. The pipette solution contained (in mM): 130 K-Gluconate, 7 KCl, 2 NaCl, 0.4 EGTA, 1 MgCl_2_, 4 ATP-Mg, 0.3 GTP-Na, 10 HEPES, 10 Tris-phosphocreatine, 10 units/ml creatine phosphokinase, pH 7.3 with KOH, 290 mOsm. Recording pipettes had <4.5 MΩ resistance. pClamp 10 (Molecular Devices) was used to acquire and analyze data. Cell capacitance and series resistance were constantly monitored throughout the recording.

### Voltage-clamp Experiments

Recording pipettes had <4.5 MΩ resistance. Series resistance (<15 MΩ) was compensated by 80%. Current traces were not leak-subtracted. Signals were filtered at 2 kHz and digitized at 20 kHz. To measure the current-voltage relationships (I-V curves) of TRESK K^+^ channels, HEK293T cells were held at −60 mV. Command steps from −100 mV to +100 mV (10 mV increments) were applied for 500 ms and then the cell was repolarized back to −60 mV. For each cell, the peak current was normalized by the membrane capacitance (a measure of cell surface area) to reflect current density.

To dissect background K^+^ currents in small-diameter (<25 µm) TG neurons, we included 1 µM tetrodotoxin (TTX) in the extracellular solution to inhibit TTX-sensitive Na^+^ currents [1], [12], [14]. Neurons were held at −60 mV and were depolarized to −25 mV for 150 ms and then the potential was ramped to −135 mV at a rate of 0.37 mV/ms every 10 sec [9], [12], [14]. We measured the outward currents at the end of the −25 mV depolarizing step. This minimized the transient voltage-gated K^+^ currents [9]. The fast TTX-resistant Na^+^ currents were also completely inactivated at the end of 150 ms depolarization [14]. Depolarization to −25 mV only evokes very small high voltage-activated Ca^2+^ currents, most of which are inactivated at the end of the 150 ms depolarization [17]. At the −60 mV holding potential, the majority of T-type Ca^2+^ channels are inactivated [18], and thus do not contribute to the currents evoked by −25 mV depolarization. To dissect currents through TRESK channels, we bath-applied 30 µM lamotrigine (Sigma) while evoking whole cell currents using this pulse protocol 12,14,19.

### Current Clamp Experiments

Neuronal excitability was studied in small-diameter TG neurons transfected with plasmids encoding mCherry and mCherry-TRESK proteins, respectively. Recording pipettes had <4.5 MΩ resistance. Series resistance (<20 MΩ) was not compensated. Signals were filtered at 10 kHz and digitized at 100 kHz. After establishing whole-cell access, membrane capacitance was determined with amplifier circuitry. The amplifier was then switched to current-clamp mode to measure V_rest_. The R_in_ was calculated by measuring the change of membrane potential in response to a 20 pA hyperpolarizing current injection from V_rest_. Neurons were excluded from analysis if the V_rest_ was higher than −40 mV or R_in_ was smaller than 200 MΩ.

To test neuronal excitability, neurons were held at V_rest_ and were injected with 1 sec depolarizing currents in 25 pA incremental steps until at least 1 AP was elicited. The rheobase was defined as the minimum amount of current to elicit at least 1 AP. The first AP elicited using this paradigm was used to measure AP threshold (the membrane potential at which dV/dt exceeds 10 V/sec), amplitude and half width. The amplitude of afterhyperpolarization (AHP) was measured from the single AP elicited by injecting a 1 ms depolarizing current in 200 pA incremental steps from the V_rest_. Data was analyzed with the Clampfit (Molecular Devices) and Origin (OriginLab) softwares.

At the end of each electrophysiological recording, neurons were incubated with FITC-conjugated isolectin B4 (FITC-IB4, 3 µg/ml) for 5 min. The FITC fluorescence on soma membrane was detected after 10 min perfusion to wash off unbound IB4. The recording pipette remained attached to the neurons during IB4 staining and washing. The V_rest_, R_in_, capacitance, series resistance and leak currents were not significantly altered after IB4 staining. None of the neurons were destroyed and/or detached from the coverslip/pipette after electrophysiological recording and/or after IB4 staining.

To test the response to capsaicin, transfected neurons were held at V_rest_ under current clamp and were recorded under gap-free mode. Cells were perfused with Tyrode solution (5 ml/min) containing 100 nM capsaicin (Sigma) for 90 sec before washing off. To record capsaicin-induced whole-cell currents, transfected neurons were held at −60 mV under voltage clamp. Cells were perfused with Tyrode solution (5 ml/min) containing 100 nM capsaicin (Sigma) for 200 sec. Capsaicin was diluted from 50 mM stock solutions (in DMSO) stored at −80°C in small aliquots and was freshly prepared on each day of recording.

### Statistical Analysis

All data are reported as mean ± standard error of the mean. The normality of each data set was assessed by χ^2^-test. Statistical significance was assessed by Fisher’s exact test, two-tailed *t*-test, one-way analysis of variance (ANOVA) with post hoc Bonferroni test, two-way repeated measures (RM) ANOVA with post hoc Bonferroni test or Kolmogorov-Smirnov test where appropriate. Differences with *p*<0.05 were considered to be statistically significant.

## Results

### The N-terminal mCherry Tag does not Affect TRESK Channel Properties in HEK293T Cells

Before expressing the mCherry-TRESK subunits in TG neurons, we compared whole-cell K^+^ currents through untagged mouse TRESK subunits and mCherry-TRESK fusion proteins in HEK293T cells. The untagged TRESK coding sequence was inserted into the expression vector upstream of the DsRed fluorescent protein coding region, separated by an internal ribosome entry site. Since the proteins are expressed on the same mRNA transcript, we used DsRed fluorescence to identify HEK293T cells expressing untagged TRESK subunits. The control group was transfected with the plasmid encoding mCherry protein, and the mCherry fluorescence was used to identify transfected cells expressing mCherry protein or mCherry-TRESK subunits.

**Figure 1:**
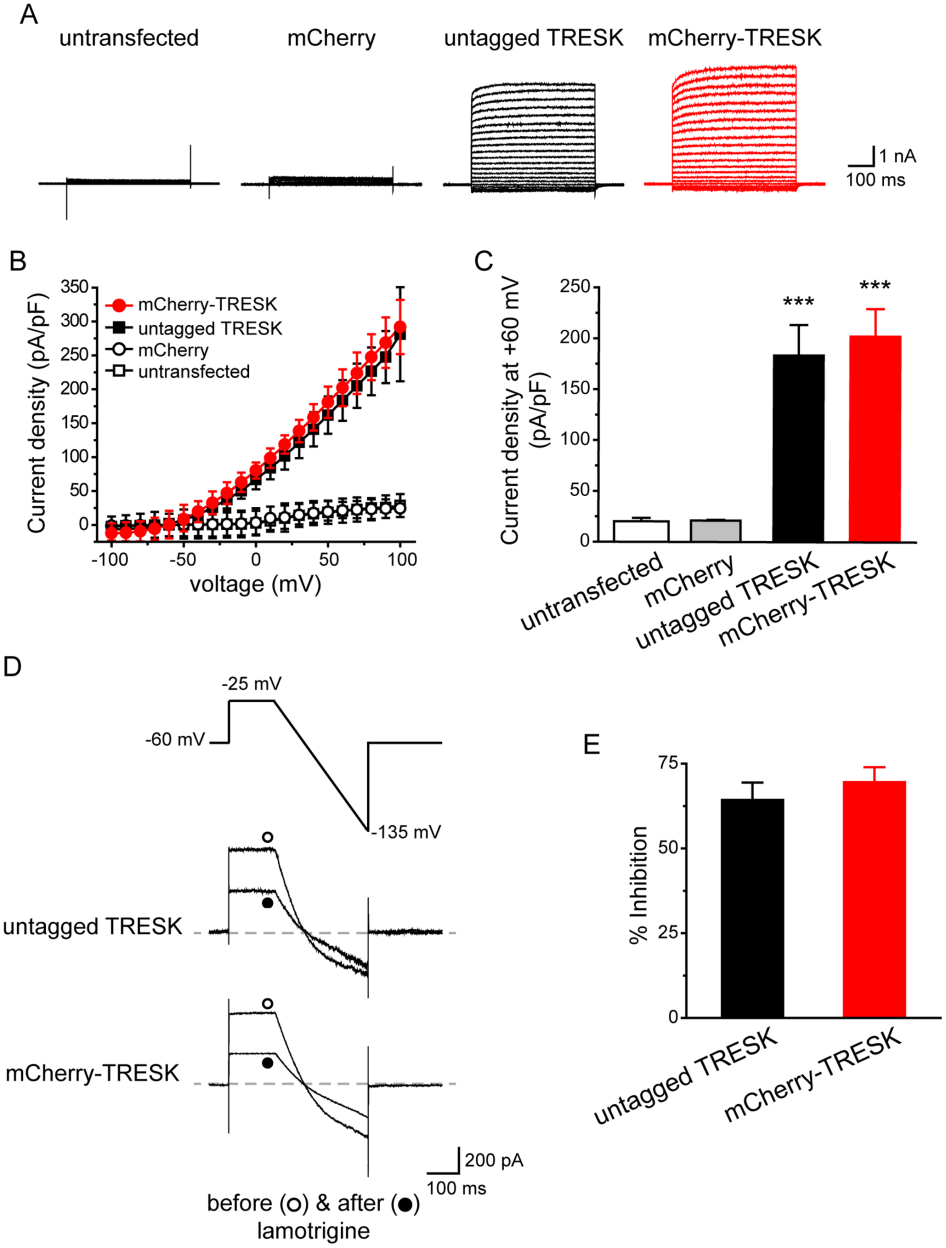
The N-terminal mCherry tag does not affect TRESK channel properties in HEK293T cells. ***A,*** Representative current records from an untransfected HEK293T cell, a cell expressing mCherry protein, a cell expressing untagged TRESK (transfected with the wtTRESK-IRES-DsRed construct) and a cell expressing mCherry-TRESK subunits, respectively. Transfected cells were held at −60 mV and were subject to 500 ms voltage steps from −100 mV to +100 mV (10 mV increments, every 10 sec) and then repolarized back to −60 mV. ***B,*** I-V curves of peak TRESK current densities in untransfected HEK293T cells and cells expressing mCherry protein, untagged TRESK and mCherry-TRESK channels, respectively (the same recording protocols as in *A*, n = 5, 8, 9 and 6 cells in each group, respectively). ***C,*** TRESK current densities at +60 mV (the same cells as in ***B***; *** *p*<0.001, one way ANOVA with post hoc Bonferroni test, compared with the untransfected group; p = 1 between the untransfected and mCherry groups; p = 1.1 between the untagged TRESK and mCherry-TRESK groups). ***D,*** Upper panel: the ramp voltage protocol used to record TRESK currents. Middle and lower panels: representative current traces from TG neurons expressing untagged TRESK and mCherry-TRESK subunits before and after the application of 30 µM lamotrigine, respectively. ***E,*** The percentage of outward current (measured at the end of the depolarizing step) inhibited by 30 µM lamotrigine (p = 0.36 between the two groups, two-tailed *t*-test; n = 6 cells in each group, respectively).

Untransfected cells and cells expressing mCherry proteins exhibited very small background leak current densities (Figure 1A–C). Cells expressing untagged and mCherry-tagged TRESK subunits both exhibited large outwardly rectifying whole-cell K^+^ currents (Figure 1A, B), consistent with previous reports [2], [4]. The two I-V curves nearly overlapped with each other (Figure 1B). In addition, the application of 30 µM lamotrigine resulted in partial inhibition of currents through both untagged and tagged TRESK channels (64±5% and 70±4%, respectively, Figure 1D, E), in line with previous studies [12], [19]. The reversal potentials of lamotrigine-sensitive currents were −79±2 mV and −82±1 mV for the untagged and tagged TRESK channels, respectively, as expected for background K^+^ channels. Taken together, we conclude that the N-terminal mCherry tag does not affect the expression level and/or other biophysical properties of the TRESK channels.

### Over-expression of TRESK Subunits Increases the Background K^+^ Currents in TG Neurons

**Figure 2:**
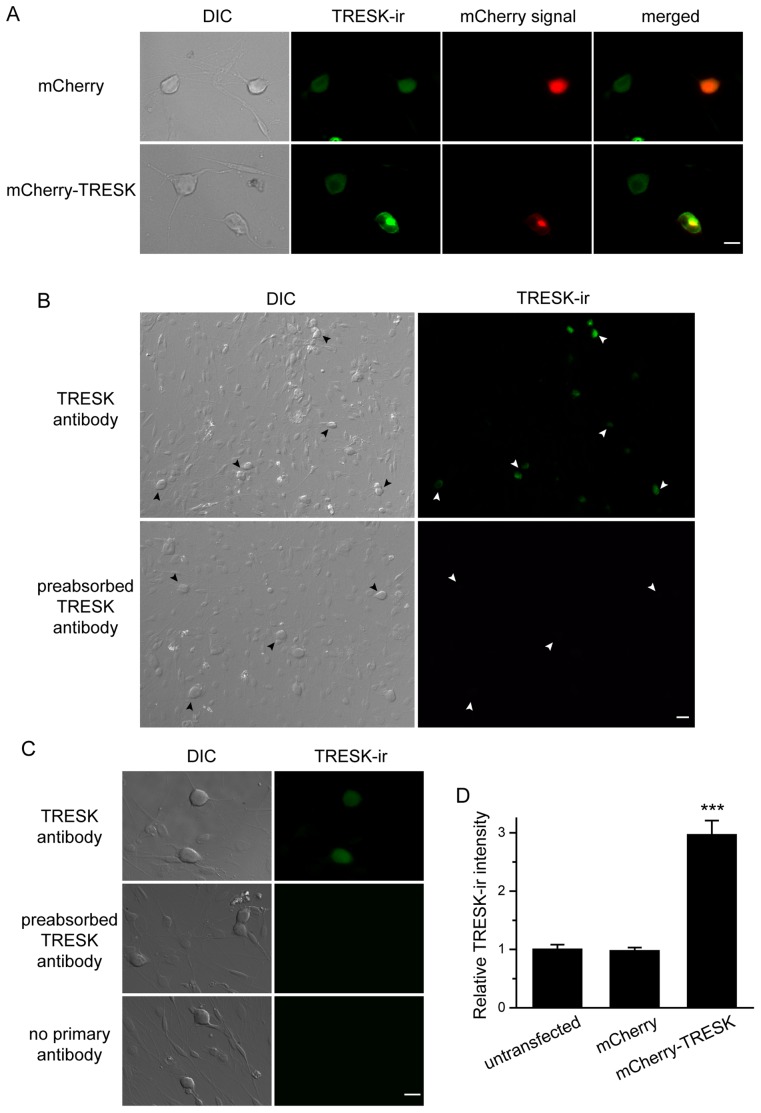
Over-expression of mCherry-TRESK subunits in cultured TG neurons. ***A,*** Representative images of cultured TG neurons expressing mCherry and mCherry-TRESK, respectively. Neurons were stained with a TRESK antibody two days post-transfection. The TRESK antibody recognizes both endogenous and over-expressed TRESK subunits. Scale bar = 10 µm. ***B,*** Low-magnification representative images of cultured TG neurons stained with the TRESK antibody (upper panel) and the same antibody pre-absorbed with antigen at 1∶10 molar ratio (lower panel). The arrowheads indicate TRESK-ir of TG neurons in the culture. Scale bar = 20 µm. ***C,*** High-magnification representative images of cultured TG neurons stained with the TRESK antibody (upper panel) and the same antibody pre-absorbed with antigen at 1∶10 molar ratio (middle panel). Lower panel shows TG neurons stained with the secondary antibody only. Scale bar = 10 µm. ***D,*** Relative TRESK-ir intensity in untransfected TG neurons, neurons expressing mCherry and mCherry-TRESK, respectively (*** *p*<0.001, one way ANOVA with *post hoc* Bonferroni test, compared with the untransfected group, n = 99, 32 and 25 cells in each group, respectively).

We expressed mCherry-TRESK subunits in cultured TG neurons from neonatal mice and monitored the mCherry fluorescence in the soma. The plasma membrane was not clearly delineated in most transfected neurons, suggesting that the majority of mCherry-TRESK proteins are localized in the intracellular organelles. Transfected neurons exhibited punctate intracellular mCherry signals in addition to diffuse, low-intensity mCherry fluorescence throughout the soma (Figure 2A). We have previously shown that the punctate signals are not mCherry-TRESK proteins misrouted to the nucleus, but likely represent newly synthesized mCherry-TRESK subunits in the intracellular organelles [14]. The control neurons expressing mCherry proteins showed bright homogenous fluorescence in the soma (Figure 2A).

To estimate the relative level of endogenous and exogenous TRESK subunits in transfected cells, we stained the TG neurons with an antibody that specifically recognizes TRESK channels in HEK293T cells [14]. Preabsorption of the antibody with antigen at 1∶10 molar ratio completely abolished the signal in cultured TG neurons (Figure 2B, C), further validating the specificity of the antibody. We found that the TRESK-ir was present in almost all TG neurons in culture (Figure 2B), in agreement with previous reports [3], [5], [9]. Non-neuronal cells had little TRESK-ir (Figure 2B, C). Quantitative analysis showed expression of mCherry protein did not alter the level of endogenous TRESK-ir (Figure 2D). In neurons expressing mCherry-TRESK, the TRESK-ir overlapped with the mCherry signal (Figure 2A), suggesting a similar subcellular localization of mCherry-TRESK and the endogenous TRESK subunits. The level of TRESK-ir in cells expressing mCherry-TRESK subunits was about 2-fold higher than that in the untransfected neurons and neurons expressing mCherry protein (Figure 2D, *p*<0.001, one way ANOVA with *post hoc* Bonferroni test).

**Figure 3:**
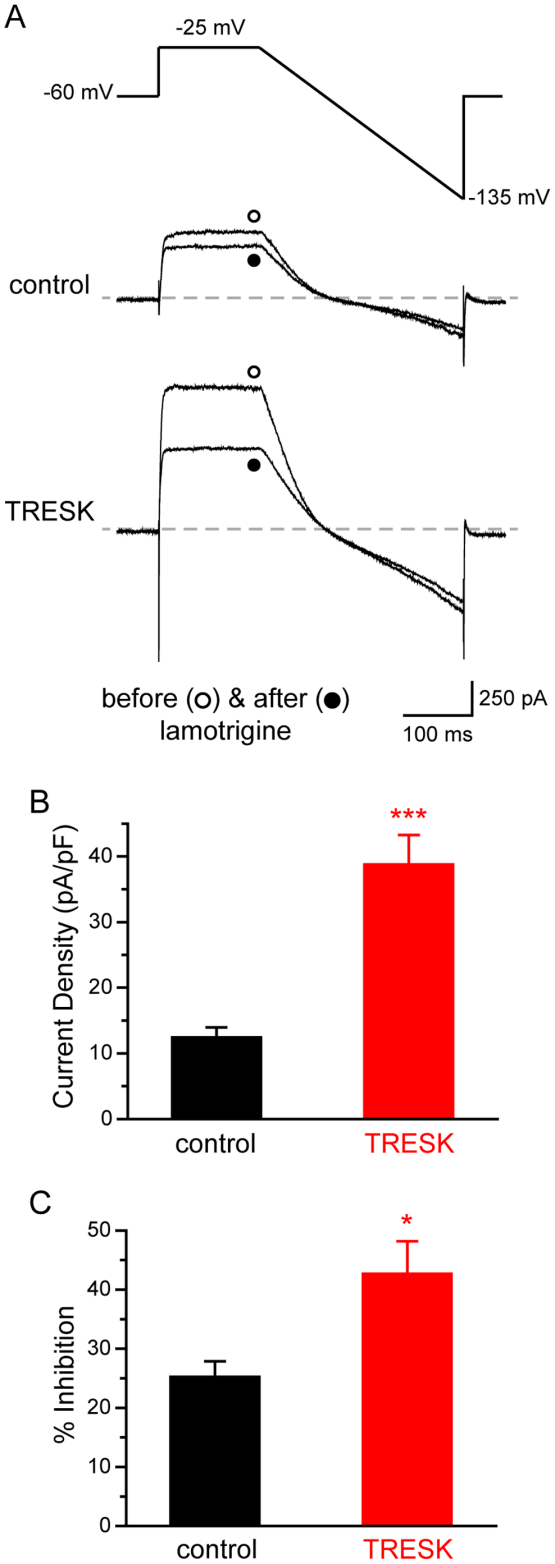
Over-expression of TRESK subunits increases the background K^+^ currents in TG neurons. ***A,*** Upper panel: the voltage protocol used to minimize transient voltage-gated K^+^, Na^+^ and Ca^2+^ currents. Middle and lower panels: representative current traces from TG neurons expressing mCherry (the control group) and mCherry-TRESK (the TRESK group) before and after the application of 30 µM lamotrigine, respectively. ***B,*** Mean total outward current density (measured at the end of the depolarizing step) in TG neurons expressing mCherry and mCherry-TRESK, respectively (*** *p*<0.001, two-tailed *t*-test, n = 22 and 21 cells in each group, respectively). ***C,*** The percentage of outward current (measured at the end of the depolarizing step) inhibited by lamotrigine in the control and TRESK groups, respectively (* *p*<0.05, two-tailed *t*-test, n = 17 and 11 cells in each group, respectively).

We proceeded to measure the size of background K^+^ currents in TG neurons expressing mCherry-TRESK subunits. We focused on the small-diameter (<25 µm) TG neurons, as the majority of neurons in this TG subpopulation are primary nociceptors [20], [21]. After blocking the TTX-sensitive Na^+^ currents with 1 µM TTX, we minimized the activation of transient voltage-gated K^+^, Na^+^ and Ca^2+^ currents by depolarizing neurons from −60 mV holding potential to −25 mV for 150 ms and subsequently hyperpolarizing neurons to −135 mV with a slow ramp (0.37 mV/ms, Figure 3A). Currents measured at the end of the depolarizing step were predominantly outward K^+^ currents. The size of the outward current was approximately 2-fold larger in neurons expressing mCherry-TRESK subunits compared with that in neurons expressing mCherry proteins (12±2 pA/pF and 39±4 pA/pF, respectively, Figure 3A–B, *p*<0.001, two-tailed *t*-test). The magnitude of current density increase corresponds well to the magnitude of TRESK-ir increase in neurons over-expressing TRESK subunits.

To dissect the currents through TRESK channels, we measured the percentage of the outward currents that was sensitive to 30 µM lamotrigine blockade. The reversal potentials of lamotrigine-sensitive currents were −81±4 mV and −80±6 mV for the control and TRESK groups, respectively. This is very close to the reversal potential of lamotrigine-sensitive currents we observed in HEK293T cells expressing TRESK channels. In the control group, 25±3% of the outward currents were inhibited by lamotrigine (Figure 3A, C). The fraction of lamotrigine-sensitive currents was significantly higher in TG neurons expressing mCherry-TRESK subunits (the TRESK group, 43±6%, *p*<0.05, two-tailed *t*-test, Figure 3A, C). Taken together, we conclude that over-expression of TRESK subunits in TG neurons increases the magnitude of TRESK currents and that, in turn, leads to an increase in the total background K^+^ currents.

### Over-expression of TRESK Subunits Reduces the Excitability of Small-diameter TG Neurons

**Figure 4:**
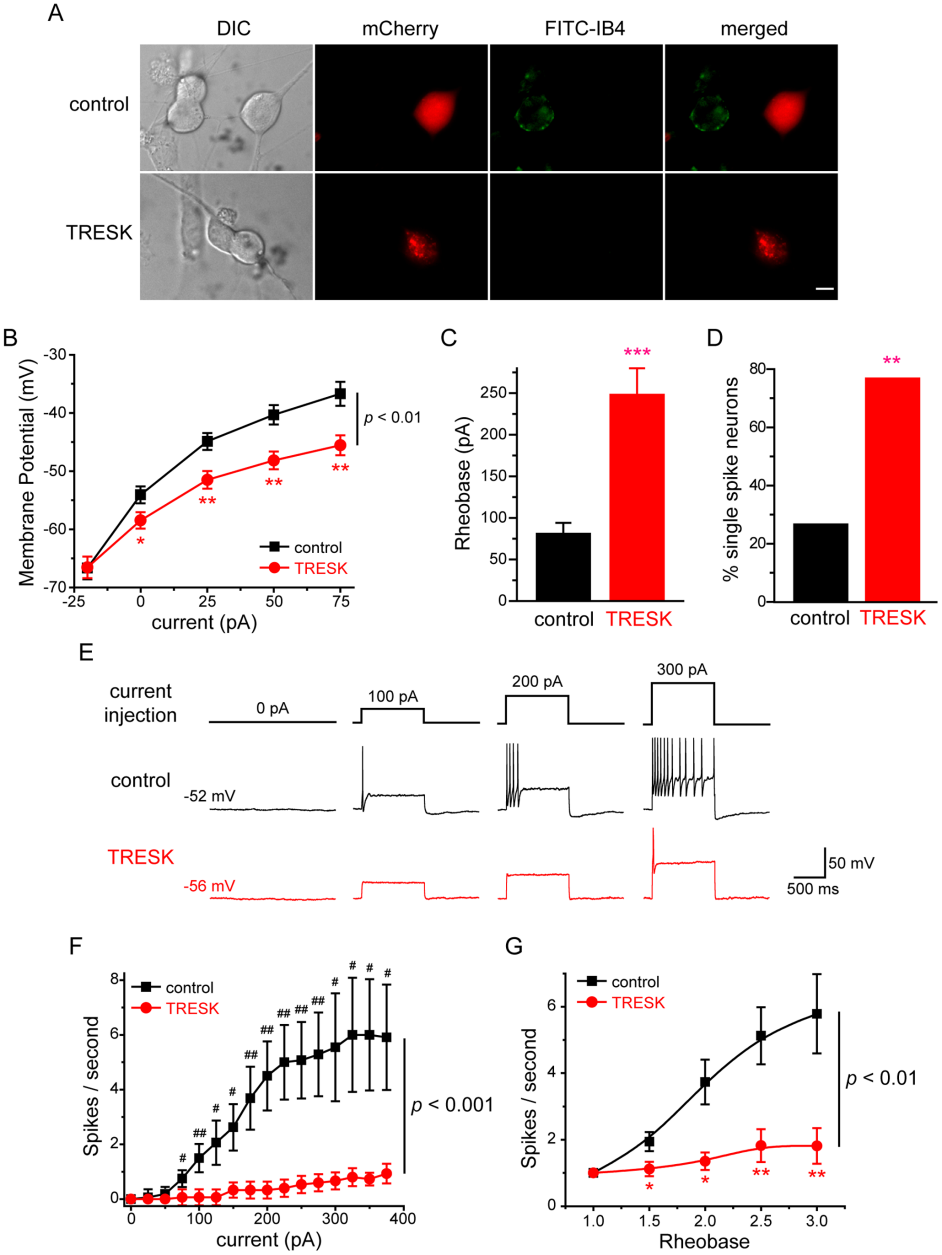
Over-expression of TRESK channels reduces the excitability of small IB4-negative TG neurons. ***A,*** Representative images of the transfected TG neurons stained with FITC-IB4. TG neurons in the control and TRESK groups were transfected with plasmids encoding mCherry protein and mCherry-TRESK subunit, respectively. Scale bar = 10 µm. ***B***, Steady-state membrane potential in response to incremental current injections in transfected, small IB4-negative TG neurons (* *p*<0.05, ** *p*<0.01 between the corresponding control and TRESK groups, two-way RM ANOVA and post hoc *t*-test with Bonferroni correction; n = 19 and 17 neurons in the control and TREK groups, respectively). ***C,*** Mean rheobase of the small IB4-negative TG neurons expressing mCherry proteins and mCherry-TRESK subunits (*** *p*<0.001, two-tailed *t*-test, same neurons as in ***B***). ***D,*** The percentage of single-spike neurons in the control and TRESK groups (* *p*<0.01, Fisher’s exact test, same neurons as in ***B***). ***E,*** Representative traces of APs generated by incremental depolarizing current injections in transfected, small IB4-negative TG neurons. The values of V_rest_ and injected current are indicated. ***F,*** The input-output plot of transfected, small IB4-negative TG neurons in the control group is significantly different from that in the TRESK group (same neurons as in ***B***, *p*<0.001, two-way RM ANOVA). The spike frequency was measured by injection of 1 sec depolarizing current in 25 pA incremental steps in each neuron (^#^ *p*<0.05, ^##^ *p*<0.01, two-tailed *t*-test between the corresponding control and TRESK groups). ***G,*** The input-output plots of the spike frequency in response to 1 sec depolarizing current injection from 1- to 3-fold rheobase in transfected, small IB4-negative TG neurons (* *p*<0.05, ** *p*<0.01 between the corresponding control and TRESK groups, two-way RM ANOVA and post hoc *t*-test with Bonferroni correction; same neurons as in ***B***).

Here, we used current clamp recording to investigate whether over-expression of TRESK subunits affects the passive and active electrophysiological properties of small-diameter TG neurons. To account for the heterogeneity of TG neurons, we further divided the small-diameter neurons based on their ability to bind to fluorescently-labeled IB4 at the end of each current clamp recording (Figure 4A, 5A). The small IB4-positive and IB4-negative primary afferent neurons exhibit distinct properties in the level of neuropeptides, the termination of central projection, the encoding of spike frequency as well as pain modalities [22]–[26].

**Figure 5:**
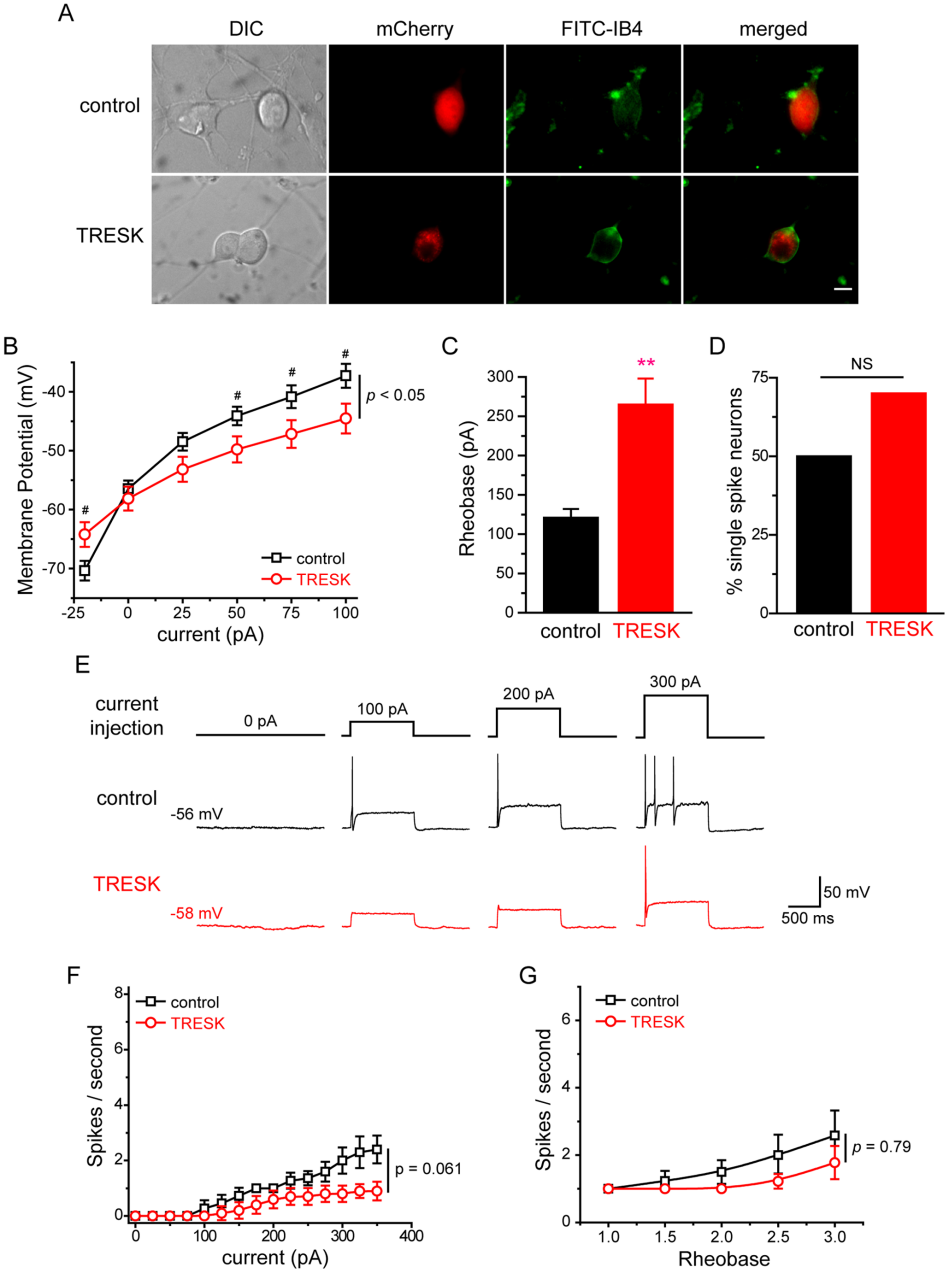
Decrease of excitability in small IB4-positive TG neurons over-expressing TRESK channels. ***A,*** Representative images of the transfected TG neurons stained with FITC-IB4. TG neurons in the control and TRESK groups were transfected with plasmids encoding mCherry protein and mCherry-TRESK subunit, respectively. Scale bar = 10 µm. ***B***, The plot of steady-state membrane potential versus injected current in the control IB4-positive TG neurons is significantly different from that in the TRESK group (*p*<0.05, two-way RM ANOVA; n = 14 and 10 neurons in the control and TREK groups, respectively). Specifically, the membrane potentials are significantly different between the two groups in response to 20 pA hyperpolarizing current injection as well as to 50∼100 pA depolarizing current injections from the V_rest_ (^#^ *p*<0.05, two-tailed *t*-test between the corresponding control and TRESK groups). ***C,*** Mean rheobase of the small IB4-positive TG neurons expressing mCherry proteins and mCherry-TRESK subunits (*** *p*<0.001, two-tailed *t*-test, same neurons as in ***B***). ***D,*** The percentage of single-spike neurons in the control and TRESK groups (p = 0.42, Fisher’s exact test, same neurons as in ***B***). ***E,*** Representative traces of APs generated by incremental depolarizing current injections in transfected, small IB4-positive TG neurons. The values of V_rest_ and injected current are indicated. ***F,*** The input-output plots of the spike frequency in response to 1 sec depolarizing current injections in 25 pA incremental steps in transfected, small IB4-positive TG neurons (*p* = 0.061 between the control and TRESK groups, two-way RM ANOVA; same neurons as in ***B***). ***G,*** The input-output plots of the spike frequency in response to 1 sec depolarizing current injection from 1- to 3-fold rheobase in transfected, small IB4-positive TG neurons (*p* = 0.79 between control and TRESK groups, two-way RM ANOVA; same neurons as in ***B***).

In small IB4-negative TG neurons, over-expression of TRESK subunits resulted in a more than 5 mV hyperpolarizing shift of the V_rest_ (Table 1 and Figure 4B), indicating that the exogenous TRESK channels contribute to the membrane conductance during resting state. Moreover, the R_in_ was significantly lower in neurons over-expressing TRESK subunits (Table 1). Consequently, injection of depolarizing currents induced smaller membrane potential changes in these neurons (Figure 4B, *p*<0.01, two-way RM ANOVA).

**Table 1:** Intrinsic properties of small-diameter TG neurons over-expressing TRESK channels.

Cell type	Diameter(µm)	Capacitance(pF)	R_in_(MΩ)	V_rest_(mV)	Rheobase(pA)	AP threshold(mV)	AP amplitude(mV)	AP half width(ms)	AHP amplitude(mV)	Cell number	
IB4-negative neurons											
Control	19.9±0.7	19.3±1.4	620±75	−53.5±1.2	88±14	−17.4±2.4	110±2	4.5±0.8	−15.5±1.1	19	
TRESK	21.8±0.8	25.0±2.8	410±60*	−58.8±1.0**	248±32***	−19.9±2.2	116±3	3.3±0.5	−14.3±1.0	17	
IB4-positive neurons											
Control	19.1±2.0	23.0±2.2	648±111	−57.0±1.1	121±11	−21.1±2.5	108±3	3.8±0.5	−13.2±1.0	14	
TRESK	22.2±0.8	25.1±2.0	304±27*	−58.1±2.0	265±33**	−19.9±2.9	116±4	3.1±0.7	−12.0±1.7	10	

Control: TG neurons expressing mCherry proteins.

TRESK: TG neurons expressing mCherry-TRESK subunits.

* *p*<0.05,

** *p*<0.01,

*** *p*<0.001, compared with the corresponding control group (two-tailed *t*-test).

Neurons in the control group express mCherry proteins. The TRESK group contains TG neurons expressing mCherry-TRESK subunits. * *p*<0.05, ** *p*<0.01 and *** *p*<0.001 compared with the corresponding control group by two-tailed *t* test.

We proceeded to investigate how over-expression of TRESK subunits alters the excitability of small IB4-negative TG neurons. Transfected neurons were held at V_rest_ and were injected with 1 sec depolarizing currents at 25 pA incremental steps to elicit APs. Consistent with the changes in passive membrane properties by the exogenous TRESK channels, we found that over-expression of TRESK subunits resulted in a more than 2-fold increase in rheobase (88±14 pA and 248±32 pA in the control and TRESK groups, respectively; Figure 4C, E and Table 1, *p*<0.001, two-tailed *t*-test), indicating that an increase in TRESK currents significantly affects AP initiation and decreases neuronal excitability. On the other hand, values of the AP threshold, amplitude, half width and AHP amplitude were all comparable between the two groups (Table 1), in line with previous studies [9], [14]. In addition, the latency to first spike was also comparable between the control and TRESK groups (13.6±1.5 ms and 13.0±1.8 ms, respectively, *p* = 0.79, two-tailed *t*-test).

Next, we tested the effect of increasing TRESK currents on spike frequency in small IB4-negative TG neurons. In neurons expressing mCherry proteins, the number of APs initially increased almost linearly in response to incremental depolarizing current injections (from 75 pA to 225 pA) and eventually reached plateau (Figure 4E, F, control group). More than 70% (14 out of 19) of the cells generated multiple spikes in response to supra-threshold current injections. The rest (26%, 5 out of 19) were single-spike neurons, i.e., they generated only one AP in response to a 1-sec depolarizing current injection from 1- to 3-fold rheobase (Figure 4D).

On the contrary, the majority of neurons over-expressing mCherry-TRESK subunits (76%, 13 out of 17) generated a single spike in response to both threshold and supra-threshold current injections (Figure 4D, *p*<0.01, Fisher’s exact test). This was also indicated by the much flatter input-output curve (Figure 4E, F, TRESK group), compared with that of the control group (*p*<0.001, two-way RM ANOVA). Similarly, the control and TRESK groups showed significant difference when we compared the number of APs elicited by a 1 sec depolarizing current injection at 1- to 3-fold rheobase (Figure 4G, *p*<0.01, two-way RM ANOVA). Taken together, we conclude that over-expression of TRESK subunits significantly reduces the excitability of small IB4-negative TG neurons, causing an increase in rheobase as well as a decrease of spike number.

We went on to examine how an increase in TRESK currents alters the excitability of small IB4-positive TG neurons. Over-expression of TRESK subunits did not alter the V_rest_ in this TG subpopulation (Table 1 and Figure 5B), but significantly reduced R_in_ (Table 1). Injection of depolarizing currents induced smaller membrane potential changes (Figure 5B, *p*<0.05, two-way RM ANOVA). Consequently, the rheobase of small IB4-positive neurons over-expressing TRESK subunits was significantly higher than that in the control group (265±33 pA and 121±11 pA, respectively; Figure 5C, E and Table 1, *p*<0.01, two-tailed *t*-test). Similar to the small IB4-negative TG population, values of the AP threshold, amplitude, half width and AHP amplitude were all comparable between the IB4-positive neurons in the control and TRESK groups (Table 1). The latency to first spike was also comparable between the two groups (12.2±1.8 ms and 11.2±2.0 ms for the control and TRESK groups, respectively, *p* = 0.86, two-tailed *t*-test). Thus, an increase in TRESK currents significantly inhibits AP initiation in both small IB4-negative and IB4-positive TG neurons.

Does over-expression of TRESK channels affect spike frequency in small IB4-positive TG neurons? Compared with the control IB4-negative TG neurons, the small IB4-positive neurons in the control group had a much flatter input-output curve (*p*<0.001, two-way RM ANOVA between control groups in Figure 4F and 5F), as 50% of them were single-spike neurons. This is consistent with the previous reports on the different spike frequency between small IB4-negative and IB4-positive primary afferent neurons [14], [23]. We found that over-expression of TRESK subunits did not significantly increase the percentage of single-spike neurons in the small IB4-positive TG population (Figure 5D, *p* = 0.42, Fisher’s exact test). Nor did it significantly decrease the spike frequency (Figure 5F, G, *p*>0.06, two-way RM ANOVA). Taken together, we conclude that over-expression of TRESK subunits reduces the excitability of small IB4-positive TG neurons mainly through increasing the current threshold for AP generation.

### Capsaicin-evoked Spikes was Reduced in TG Neurons Over-expressing TRESK Channels

**Figure 6:**
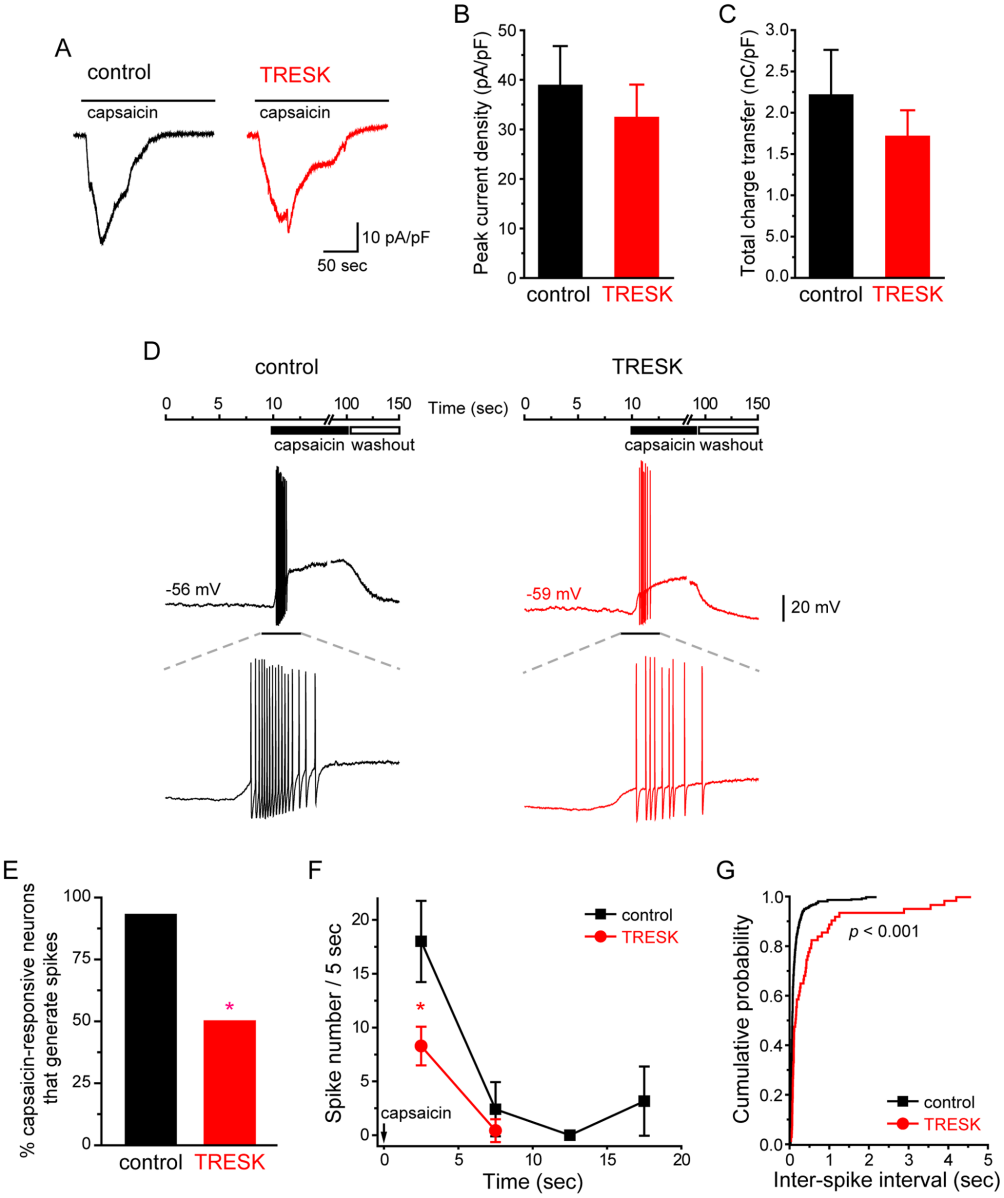
Over-expression of TRESK channels reduces capsaicin-evoked spikes in TG neurons. ***A,*** Representative traces of whole-cell currents induced by capsaicin (100 nM) in transfected, small-diameter TG neurons. TG neurons in the control and TRESK groups were transfected with plasmids encoding mCherry protein and mCherry-TRESK subunit, respectively. ***B,*** Peak capsaicin-evoked current density of TG neurons in the control and TRESK groups (*p* = 0.51, two-tailed *t*-test, n = 6 and 8 cells in each group, respectively). ***C,*** Mean charge transfer of the control and TRESK groups of neurons in response to 200 sec application of capsaicin (*p* = 0.41, two-tailed *t*-test, same neurons as in ***B***). For each cell, the charge transfer was normalized by the membrane capacitance to correct for the cell surface area. ***D,*** Representative traces of membrane depolarization and APs generated by capsaicin (100 nM) in transfected, small-diameter TG neurons. The values of V_rest_ of each neuron are indicated. The lower panel shows traces on an expanded timescale for the timeframe indicated by the horizontal bar above. ***E,*** The percentage of capsaicin-responsive neurons in the control and TRESK groups that also exhibited capsaicin-evoked APs (* *p*<0.05, Fisher’s exact test). ***F,*** The number of capsaicin-evoked spikes/5 sec in the control and TRESK groups (* *p*<0.05, two tailed *t*-test; n = 14 and 6 neurons in each group, respectively). Arrow indicates the onset of capsaicin application (time 0). ***G,*** Cumulative probabilities of inter-spike intervals of capsaicin-evoked APs in control and TRESK groups (** *p*<0.001, Kolmogorov-Smirnov test; n = 337 spikes/14 neurons and 63 spikes/6 neurons, respectively; same neurons as in ***C***).

Does over-expression of TRESK subunits attenuate the response of TG neurons evoked by noxious stimuli? To address this question, we compared capsaicin-evoked spikes in TG neurons expressing mCherry protein and mCherry-TRESK subunits, respectively. Capsaicin selectively activates the transient receptor potential vanilloid receptor 1 (TRPV1) in a subpopulation of small-diameter TG neurons, resulting in membrane depolarization and, in some cases, multiple spikes [27]–[30]. First, we found that the percentage of neurons that were depolarized by bath application of 100 nM capsaicin was similar in the control and TRESK groups (40% and 30%, respectively; *p* = 0.35, Fisher’s exact test; n = 37 and 40 neurons, respectively). The magnitude of capsaicin-induced membrane depolarization was also comparable between the two groups (24±8 mV and 24±3 mV, respectively). In addition, we measured the capsaicin-induced currents in transfected neurons (Figure 6A). Both the peak current density and the total charge transfer in response to 200 sec capsaicin application were comparable between the control and TRESK groups of neurons (Figure 6B, C), suggesting that the increase in TRESK channel activity did not compromise the expression and function of TRPV1 channels in capsaicin-responsive neurons.

Next, we quantified the APs evoked by 90 sec application of 100 nM capsaicin (Figure 6D). In the control group, all but one capsaicin-responsive neurons generated multiple spikes in response to capsaicin (93%, 14 out of 15 cells). Conversely, in TG neurons over-expressing TRESK subunits, we observed spikes in only 50% of capsaicin-responsive cells (6 out of 12, *p*<0.05, compared with the control group, Fisher’s exact test, Figure 6E). The other 6 neurons were depolarized by capsaicin but failed to generate AP. The latency to first spike was comparable between the control and TRESK groups (0.55±0.11 sec and 0.79±0.14 sec, respectively, *p* = 0.17, two-tailed *t*-test). Most of the capsaicin-evoked APs occurred during the first 5 sec of capsaicin application (Figure 6F). Capsaicin induced twice as many spikes in control neurons than in neurons over-expressing TRESK subunits (Figure 6F, 18±3 spikes and 8±2 spikes upon 5 sec capsaicin treatment, respectively; *p*<0.05, two-tailed *t*-test). The inter-spike intervals of capsaicin-induced APs were significantly shorter in the control group compared with that in the TRESK group (Figure 6G, *p*<0.001, Kolmogorov-Smirnov test). These results indicate that an increase in TRESK channel activity effectively attenuates capsaicin-induced excitation of TG neurons by reducing the number of APs as well as lowering the spike frequency.

## Discussion

That TRESK K^+^ channel may be a potential target for novel pain medicine has been suggested by recent genetic, anatomical and functional studies on the contribution of TRESK channels to the excitability of primary afferent neurons in acute and chronic pain states [1], [3], [5], [7], [9], [11]–[16]. Here, we directly tested this possibility using cultured TG neurons from neonatal mice as a platform. Since a TRESK-specific channel opener is not currently available, we mimicked its effects by over-expressing mCherry-tagged TRESK subunits in small-diameter TG neurons. This resulted in a 2-fold increase in the level of TRESK subunits and a significant increase in the fraction of lamotrigine-sensitive persistent K^+^ currents as well as the size of total background K^+^ currents in these neurons. Consequently, over-expression of TRESK subunits reduced R_in_ in both IB4-positive and IB4-negative TG neurons, causing smaller membrane potential changes in response to depolarizing current injection. This is consistent with our previous report that the activation of endogenous TRESK channels provides feedback control of membrane depolarization in TG neurons [14]. It has also been shown that endogenous TRESK channels are not involved in controlling the V_rest_ in primary afferent neurons [9], [14]. Interestingly, over-expression of TRESK subunits lead to a hyperpolarizing shift of V_rest_ in IB4-negative TG neurons but not the IB4-positive population. One caveat is that over-expression of TRESK subunits for 2–4 days may cause compensatory changes in other ion channels in TG neurons. It will be interesting to test whether and how acute inhibition or activation of endogenous TRESK channels would alter V_rest_ and R_in_ in primary afferent neurons when TRESK-specific blockers and openers become available in the future.

A major finding of this study is that over-expression of TRESK subunits substantially reduces the excitability of small-diameter TG neurons. In both IB4-positive and IB4-negative TG population, the current threshold (rheobase) to induce AP was significantly increased, from 80∼120 pA in control neurons to around 250 pA in neurons expressing mCherry-TRESK. Moreover, the spike frequency in response to supra-threshold stimuli was greatly decreased in IB4-negative neurons over-expressing TRESK subunits. In fact, an increase in TRESK channel activity is sufficient to transform the majority of small IB4-negative neurons from multiple-spike neurons to single-spike neurons. On the other hand, our results and previous studies both show that small IB4-positive primary afferent neurons exhibit lower spike frequency than IB4-negative neurons [14], [23]. Consequently, over-expression of TRESK channels did not further reduce the spike frequency in this TG subpopulation. These results, along with previous reports on the hyper-excitability of primary afferent neurons as the result of TRESK channel dysfunction [9], [12]–[14], indicate that there is an inverse correlation between the endogenous TRESK channel activity and neuronal excitability. Both TRESK channel opener and inhibitor would affect the excitability of primary afferent neurons, although in the opposite way.

Another important issue we have addressed is whether TRESK modulates the response of TG neurons to a noxious stimulus. TRPV1, a non-selective cation channel, is expressed in a subpopulation of primary afferent nociceptors and is activated by a variety of endogenous and exogenous stimuli, including heat, acidic pH and capsaicin, the active ingredient of hot chili peppers [27], [31]. It is well established that TRPV1 contributes to the detection and integration of noxious chemical and thermal stimuli in both normal and chronic pain states [31]. Here we showed that capsaicin induced strong depolarization and a burst of spikes in control TG neurons, similar to what was found in a previous study [28]. Conversely, the same dose of capsaicin evoked fewer numbers of APs with longer inter-spike intervals in neurons over-expressing TRESK subunits. Moreover, in a significant fraction of those neurons, capsaicin only induced depolarization but failed to generate APs. Taken together, these results indicate that enhancing the TRESK channel activity may effectively attenuate the responsiveness of primary afferent neurons to noxious stimuli. Admittedly, these experiments were conducted in transfected, cultured TG neurons from neonatal mice. Given the limitation of the experimental system, our data should be interpreted with caution. On the other hand, our results are supported by previous studies showing that over-expression of TRESK subunits in DRG neurons inhibits capsaicin-evoked substance P release and attenuates nerve injury-induced mechanical allodynia [15], [16].

Dysfunction of TRESK channels results in hyper-excitation of primary afferent neurons; thereby contributing to chronic neuropathic pain and migraine headache, both of which are highly debilitating diseases and in dire need of more effective treatments [3], [12]–[14]. The TRESK channel offers a highly desirable target for the development of new analgesic drugs. TRESK is more abundantly expressed in TG and DRG neurons than other tissues [3]; whereas within primary afferent neurons, the expression pattern is relatively uniform. Thus, modulation of TRESK activity is expected to alter the excitability of many TG and DRG neurons and to exhibit minimal effect on other tissues. Since TG and DRG lie outside the blood-brain barrier, it is possible to selectively modulate TRESK activity in primary afferent neurons, leaving TRESK channels in the central nervous system unperturbed. Recent progress in structure and function studies of TRESK and other K_2P_ channels would facilitate the development of potent and selective TRESK channel openers as potential analgesic drugs [7], [32]–[36].

In summary, we have observed that over-expression of TRESK subunits results in an increase in background K^+^ currents, a decrease of R_in_, and ultimately a significant decrease in the excitability of small-diameter TG neurons. Importantly, over-expression of TRESK subunits inhibits capsaicin-evoked spikes in TG neurons, suggesting that a TRESK-specific channel opener may exhibit analgesic effect via reducing the excitability of primary afferent neurons.
